# Correlation Analysis between Residual Pain after Vertebral Augmentation and the Diffusion Distribution of Bone Cement: A Retrospective Cohort Study

**DOI:** 10.1155/2023/1157611

**Published:** 2023-01-04

**Authors:** Kang Yao, Yungang Chen, Xiaoying Wang, Qianqian Yao, Kaiying Cui, Wenming Chen, Pengfei Hou, Ning Yu, Zhenyue Zhang, Wenxin Gao, Haipeng Xue, Yanke Hao

**Affiliations:** ^1^The First Clinical Medical School, Shandong University of Traditional Chinese Medicine, Jinan, Shandong 250014, China; ^2^Shandong University of Traditional Chinese Medicine, Jinan, Shandong 250014, China; ^3^Jinan Vocational College of Nursing, Jinan, Shandong 250014, China; ^4^Shandong Provincial Third Hospital, Jinan, Shandong 250031, China; ^5^Affiliated Hospital of Shandong University of Traditional Chinese Medicine, Jinan, Shandong 250014, China

## Abstract

**Objective:**

To explore the influence and potential factors of the bone cement dispersion state on residual pain after vertebral augmentation.

**Methods:**

The cases included in this retrospective cohort study were patients treated with vertebral augmentation (VA) for osteoporotic vertebral compression fractures (OVCFs) between July 2018 and June 2021. According to the type of cement diffusion distribution, the patients were divided into a sufficient diffusion group (Group A) and an insufficient diffusion group (Group B). The differences in the baseline data, visual analog scale (VAS), Oswestry disability index score (ODI), injured vertebral height (IVH), and local kyphosis angle (LKA) between the two groups were analyzed. Assessments were performed preoperatively on the 2nd day postoperation and at the last follow-up. The imaging data of injured vertebrae were accurately reconstructed by a GE AW4.7 workstation, and the differences in the vertebral body volume, bone cement volume, and bone cement volume ratio were compared between the groups.

**Result:**

After screening, 36 patients were included. (1) The postoperative VAS and ODI scores of the two groups were significantly improved compared with the preoperative scores. (2) On the 2nd day postoperation and the last follow-up, the VAS and ODI scores of Group A were significantly different from those of Group B, and Group A outperformed Group B. (3) The IVH and LKA of the two groups were improved after the operation, and no significant difference was found between the groups. (4) Significant differences were found in the bone cement volume and bone cement volume ratio between the groups, and Group A was larger than Group B.

**Conclusions:**

Sufficient bone cement diffusion can reduce residual pain after vertebral augmentation.

## 1. Introduction

Osteoporotic vertebral compression fractures (OVCFs) occur secondary to osteoporosis [1]. Osteoporosis is characterized by decreased bone mass and bone microstructure destruction, increasing the risk of fragility fractures. OVCFs are one of the most severe fracture types, accounting for approximately 50% of osteoporotic fractures, and have become the third most common fragility fracture worldwide [2, 3]. OVCFs are commonly found in the elderly and can lead to chronic pain, motor disorders, spinal deformity, decreased quality of life and increased mortality, seriously affect human physical health and quality of life, and place a heavy burden on families and society [4–6]. Currently, conservative treatment usually adopts bed rest, nonsteroidal anti-inflammatory drugs, antiresorptive medications, external fixed braces, traditional Chinese medicine functional reduction, and other methods [7]. However, conservative treatment increases the risk of bone nonunion, bedsores, venous thromboembolism, persistent pain, and spinal deformity [8, 9]. Therefore, vertebral augmentation (VA) has become a widely accepted, safe, and effective treatment method because of its minimal trauma, short operation time, rapid pain relief, partial restoration of vertebral height, and effective increase in vertebral stability [10, 11].

Although VA is the main surgical method for treating OVCFs and has many advantages, residual pain after surgery has always been a challenge for doctors and patients [12, 13]. Many reasons can account for postoperative residual pain, such as osteoporosis, vertebral degeneration, vertebral instability, vertical spinal muscle spasm, thoracolumbar fascia injury, intraoperative injury, vertebral infection, and mental factors [14–17]. Recently, the diffusion distribution of cement in the injured vertebra was found to affect treatment [18, 19]. However, the correlation between intraoperative cement dispersion and postoperative residual pain has not been clarified, and no literature clearly indicates the relationship between the degree of cement dispersion and residual pain. We hypothesize that when the diffusion distribution of the cement in the vertebral body is insufficient, the characteristics of the cement itself cannot be fully developed, and it cannot provide effective biological stability for the vertebral body and ultimately cannot achieve analgesia, resulting in postoperative residual pain.

Therefore, we retrospectively analyzed the case data of OVCFs treated with VA, set the diffusion distribution status of bone cement within the fracture line as an independent variable, and evaluated the variability in the visual analog scale (VAS), Oswestry disability index score (ODI), injured vertebral height (IVH), and local kyphosis angle (LKA) at different times after surgery. In addition, to analyze the potential influencing factors of residual pain, we used the GE AW4.7 workstation to model the patient imaging data accurately and measure the volume of injured vertebra, cement volume, and cement volume ratio (the ratio of cement volume to vertebral volume). Furthermore, we comprehensively analyzed the effect of the bone cement diffusion distribution status on postoperative residual pain.

## 2. Methods

This retrospective cohort study comprised patients with OVCFs treated at the Affiliated Hospital of Shandong University of Traditional Chinese Medicine from July 2018 to June 2021. The study was approved by the Ethics Committee of the Affiliated Hospital of Shandong University of Traditional Chinese Medicine (2022-104-KY).

### 2.1. Inclusion Criteria

The inclusion criteria include the following: ① patients with a clear medical history, clinically diagnosed with OVCFs, and with a single vertebral fracture of the thoracic or lumbar spine subjected to conservative treatment for 2–6 weeks that was ineffective; ② patients with X-ray films showing wedge-shaped and flat vertebral changes of the thoracic or lumbar vertebral bodies; ③ patients with radiologically confirmed fresh compression fracture, magnetic resonance imaging (MRI) showing edema, or an X-ray/computed tomography (CT) scan proven fracture not older than 3 months, without neurospinal compression symptoms; ④ patients treated with unilateral puncture balloon kyphoplasty (BKP); ⑤ patients with no diseases affecting bone metabolism such as abnormal parathyroid function.

### 2.2. Exclusion Criteria

The exclusion criteria include the following: ① patients with incomplete imaging and medical records and a follow-up time of less than 12 months; ② patients with other neurovascular injuries or serious cardiovascular diseases; ③ patients with pathological fractures caused by the long-term use of hormones or tumors; ④ patients with spinal metastases; ⑤ patients with postoperative complications such as bone cement leakage, nerve compression, vascular embolism, infection, and pulmonary embolism.

### 2.3. Surgical Criteria

Surgical method: percutaneous kyphoplasty. The surgery was performed by the same trained team of physicians in strict compliance with the procedures. The details are as follows: the patient is in the prone position, C-arm fluoroscopy is used to determine and mark the needle entry point of the injured vertebra (right side of injured vertebra, outside of pedicle), the operation area is routinely disinfected, and 1% lidocaine is used for local anesthesia. Under fluoroscopy, a puncture needle was used to puncture the middle and posterior 1/3 of the injured vertebral body, the needle core was withdrawn, and the guide drill was rotated into the anterior and middle 1/3 of the vertebral body through the puncture channel, anteroposterior to the spinous process. The pilot drill was withdrawn, the balloon was inserted, and the balloon was gradually pressurized and maintained at 10 atm. After observing the recovery of the height of the injured vertebra, the balloon was removed. The bone cement (Spine PMMA; OSTEOPAL® V; Heraeus Medical GmbH, Germany) was adjusted to the toothpaste stage and slowly injected into the vertebral body through the puncture channel under fluoroscopy. The filling degree of the bone cement and presence or absence of leakage were observed by fluoroscopy. After the bone cement hardened, the puncture needle was slowly removed, and the needle hole was covered with a sterile dressing.

### 2.4. Group Formation

**Figure 1:**
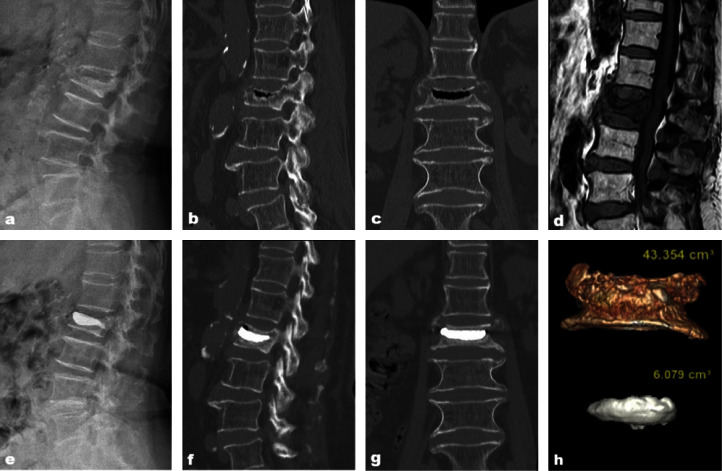
An 80-year-old female patient is diagnosed with an L2 osteoporotic vertebral compression fracture (OVCF). (a) Preoperative lateral X-ray. (b) Preoperative sagittal CT. (c) Preoperative coronal CT. (d) Preoperative MRI. (e) Postoperative lateral X-ray. (f) Postoperative sagittal CT. (g) Postoperative coronal CT. (h) 3D image of the vertebral body and bone cement.

**Figure 2:**
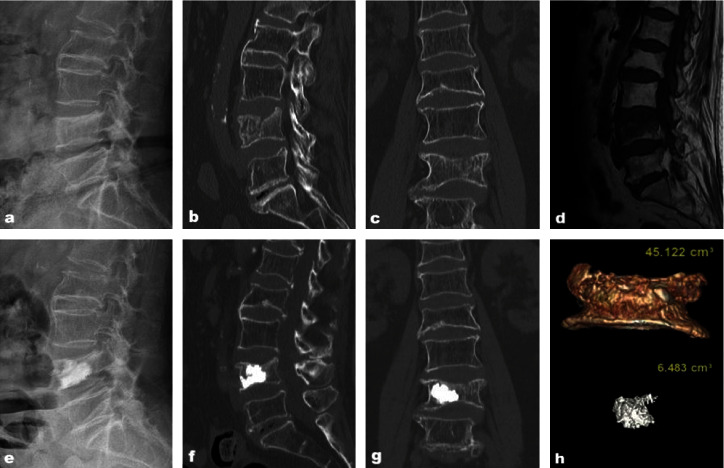
An 80-year-old female patient is diagnosed with an L4 osteoporotic vertebral compression fracture (OVCF). (a) Preoperative lateral X-ray. (b) Preoperative sagittal CT. (c) Preoperative coronal CT. (d) Preoperative MRI. (e) Postoperative lateral X-ray. (f) Postoperative sagittal CT. (g) Postoperative coronal CT. (h) 3D image of the vertebral body and bone cement.

Based on the inclusion and exclusion criteria, we screened cases meeting the criteria from the hospital electronic medical record system for a total of 36 cases. The time span was three years. This study comprised two groups, Group A with sufficient diffusion (Figure 1) and Group B with insufficient diffusion (Figure 2). Referring to the evaluation method of the diffusion status of bone cement within the fracture line, cases were assigned to the corresponding group after reaching a consensus by three experienced physicians based on the imaging data. Finally, Group A included 23 cases, and Group B included 13 cases.

The evaluation method for the degree of diffusion within the bone cement fracture line are as follows: first, the fracture line location was determined preoperatively. The CT sagittal view showed that the fracture line was band-like, with compressed dense shadows or vacuum fissures. The position of the cortical fissure around the vertebral body was observed to determine the specific direction of the fracture line (Figures 1(a)–1(d) and 2(a)–2(d)). Then, the degree of bone cement dispersion was observed postoperatively. Referring to the specific location of the fracture line determined before the operation, the X-ray films were observed in the frontal, lateral or coronal, and sagittal views of CT after the operation. If the bone cement fully covered the fracture line, it was considered to have good diffusion, and if it did not fully cover the fracture line, it was considered to have poor diffusion (Figures 1(e)–1(g) and 2(e)–2(g)).

### 2.5. Primary Outcomes

The primary outcomes included the VAS scores and ODI scores in the patients at preoperation, 2nd day postoperation, and last follow-up. Both scores were extracted from the medical record system. The VAS score ranges from 0 to 10; the higher is the score, the more severe is the pain. The ODI score ranges from 0 to 100; the higher is the score, the more severe is the dysfunction. Those scores are recorded by the patient according to their own conditions.

### 2.6. Secondary Outcomes

Secondary outcomes included the following indicators: ① baseline patient data, including age (years), sex, bone mineral density (BMD, g/cm³), imaging data (X-ray, MRI, CT), surgery time (min), and bone cement injection volume (ml); ② IVH (mm, lateral X-ray, the distance between the upper terminal lamina and the lower terminal lamina) and LKA (degrees, angle between the superior endplate from the vertebral body one level above the injured vertebral body and the inferior endplate of the vertebral body one level below); ③ postoperative vertebral body volume (cm³), bone cement volume (cm³), and bone cement volume ratio (%) calculated by the GE AW4.7 workstation.

The GE AW4.7 workstation processed the Digital Imaging and Communications in Medicine (DICOM) images of thin-slice CT scans using 3D functions to calculate the bone cement and vertebral volumes. Thin-layer CT images of the patient 2 d after surgery were extracted using the following scanning parameters: voltage, 120 kV; current smart mA, 200–500 mA; matrix, 512 × 512 pixels; scanning slice thickness, 0.625 mm. The imaging data were stored in the DICOM format. The images were transferred to the GE AW4.7 workstation, and the soft tissue thin-layer sequence was selected in the software and entered into the VR mode for volume reconstruction. The soft tissue thin-layer sequence was selected in the software, the VR mode was entered, and volume reconstruction was performed. scalpel, add, remove, and pick from VR and other functions were used to finely adjust the vertebral body, bounded by the upper and lower cuts of the pedicle, to retain the diseased vertebra, form a separate fracture vertebral model and measure the vertebral volume. Then, the bone cement was separated from the diseased vertebra in the software, and a high-qualitythree-dimensional model of the bone cement was obtained using the three-dimensional reconstruction function. The volume of the bone cement was measured, and the volume ratio of the bone cement was calculated (Figures 1(h) and 2(h)).

### 2.7. Statistical Methods

Statistical analysis was performed using IBM SPSS Statistics 26.0 (version number: R26.0.0.064, IBM, USA). Count data such as sex were expressed as counts (percentage) (*n* (%)), where *n* represents the sample size, using the chi-squared test. The variables data, including age, BMD, surgery time, bone cement injection volume, VAS, ODI, postoperative vertebral body volume, bone cement volume, bone cement volume ratio, IVH, and LKA, were assessed in accordance with normal distribution and homogeneity of variance and were expressed as means ± standard deviation (SD). Between-group comparisons were performed using independent sample *t*-test, within-group comparisons were performed using paired sample *t*-test, and 95% confidence intervals (CIs) were calculated. *P* values <0.05 indicated a significant difference, *P* values <0.01 indicated a very significant difference, and *P* values >0.05 indicated a nonsignificant difference. *P* values <0.05 were considered statistically significant differences.

## 3. Results

### 3.1. Baseline Characteristics

A total of 36 patients with OVCFs were included in this study (Supplementary file 1), all with thoracic or lumbar single vertebral fractures—23 patients in Group A and 13 patients in Group B. The fracture segments were concentrated in the thoracolumbar segments, with 16 patients in Group A and 9 patients in Group B. The age (76.30 ± 9.88 vs. 74.38 ± 7.29; 95% CI: −4.463 to 8.303; *P*=0.545), sex (*P*=0.274), BMD (−2.84 ± 0.32 g/cm³ vs. −2.93 ± 0.41 g/cm³; 95% CI: −0.161 to 0.336; *P*=0.480), injection volume (6.11 ± 1.65 ml vs. 5.15 ± 1.88 ml; 95% CI: −0.268 to 2.177; *P*=0.122), and surgery time (30.87 ± 8.02 min vs. 33.46 ± 8.43 min; 95% CI: −8.351 to 3.167; *P*=0.367) were not significantly different between the groups (Table 1).

### 3.2. VAS and ODI

**Figure 3:**
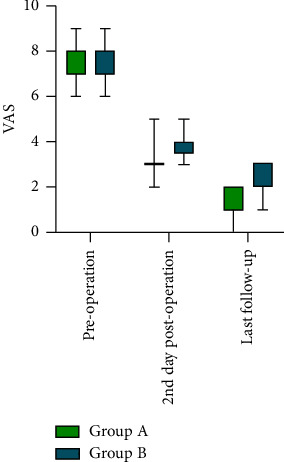
VAS between the groups.

**Figure 4:**
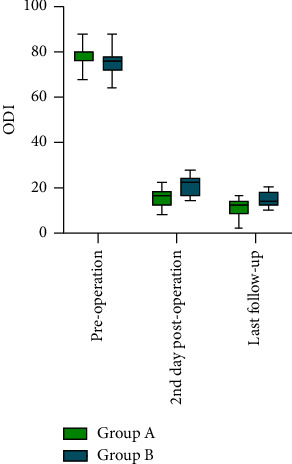
ODI between the groups.

Comparison between the groups is shown in Table 1. Regarding VAS (Figure 3), no significant difference was found between preoperative Group A and Group B. However, on the 2nd day postoperation, Group A was lower than Group B (3.17 ± 0.65 vs. 3.85 ± 0.56; 95% CI: −1.108 to −0.236; *P*=0.004). At the last follow-up, Group A was significantly lower than Group B (1.35 ± 0.57 vs. 2.31 ± 0.75; 95% CI: −1.412 to −0.508; *P* < 0.001). Similarly, no significant difference was found between preoperative Group A and Group B regarding the ODI (Figure 4). On the 2nd day postoperation, Group A was lower than Group B (15.48 ± 4.14 vs. 20.77 ± 4.67; 95% CI: −8.346 to −2.236; *P*=0.001). At the last follow-up, Group A was significantly lower than Group B (10.52 ± 3.78 vs. 14.77 ± 3.52; 95% CI: −6.847 to −1.648; *P*=0.002).

Within-group comparison of Group A is shown in Table 2. The VAS was 7.13 ± 0.76 before surgery and decreased to 3.17 ± 0.65 on the 2nd day postoperation (paired 1, 95% CI: 3.713–4.200; *P* < 0.001) and had already decreased to 1.35 ± 0.57 at the last follow-up (paired 2, 95% CI: 1.421–2.231; *P* < 0.001). The ODI decreased to 77.65 ± 4.58 before surgery, to 15.48 ± 4.14 on the 2nd day postoperation (paired 1, 95% CI: 15.48 ± 4.14; *P* < 0.001), and to 10.52 ± 3.78 at the last follow-up (paired 2, 95% CI: 2.990–6.923; *P* < 0.001).

Within-group comparison of Group B is shown in Table 2. The VAS was 7.31 ± 0.86 before surgery and decreased to 3.85 ± 0.56 on the 2nd day postoperation (paired 1, 95% CI: 2.992–3.931; *P* < 0.001) and had already decreased to 2.31 ± 0.75 at the last follow-up (paired 2, 95% CI: 1.008–2.068; *P* < 0.001). The ODI decreased to 75.23 ± 5.75 before surgery, to 20.77 ± 4.66 on the 2nd day postoperation (paired 1, 95% CI: 50.572–58.351; *P* < 0.001), and to 14.77 ± 3.52 at the last follow-up (paired 2, 95% CI: 2.546–9.454; *P*=0.003).

The VAS and ODI changes in the overall sample (*n* = 36) were observed using paired sample *t*-test, both showing *P* < 0.001, with a statistically significant difference (Table 3).

### 3.3. Secondary Outcomes

**Figure 5:**
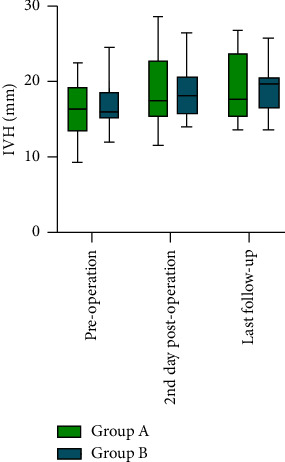
IVH (mm) between the groups.

**Figure 6:**
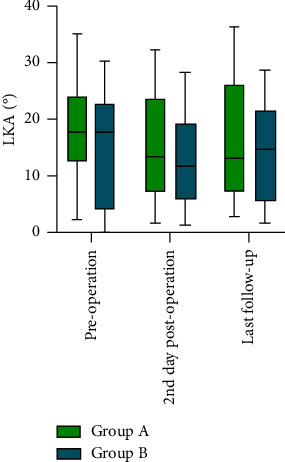
LKA (°) between the groups.

The secondary outcomes are presented in Table 1. On the 2nd day postoperation, the IVH was increased in both groups compared with that preoperatively (Figure 5), and no significant difference was found in the comparison between Group A and Group B (18.74 ± 4.56 mm vs. 18.83 ± 3.75 mm; 95% CI: −3.121 to 2.933; *P*=0.950). Regarding the LKA (Figure 6), both groups were reduced compared with before surgery, and no significant difference was found between Group A and Group B (14.89° ± 8.76° vs. 13.17° ± 7.99°; 95% CI: −4.269 to 7.711; *P*=0.563). At the last follow-up, IVH was not significant (18.87 ± 4.26 mm in Group A and 18.87 ± 3.47 mm in Group B; 95% CI: −2.822 to 2.818; *P*=0.999). The LKA increased again, rising to 16.11 ± 9.65 in Group A and 14.28 ± 8.68 in Group B (95% CI: −4.739 to 8.408; *P*=0.574).

Concerning the vertebral body volume, no significant difference was found between Group A and Group B (29.86 ± 7.99 cm³ vs. 33.81 ± 9.27 cm³; 95% CI: −9.921 to 2.016; *P*=0.187). Regarding the bone cement volume, Group A was larger than Group B (7.08 ± 2.25 cm³ vs. 5.14 ± 1.60 cm³; 95% CI: 0.501 to 3.381; *P*=0.010), and the difference was statistically significant. The volume ratio of Group A (23.70%) was greater than that of Group B (15.11%), with *P* < 0.001, and the difference was statistically significant.

## 4. Discussion

### 4.1. Vertebral Augmentation Can Reduce Pain in Patients with OVCFs

The study findings showed that the postoperative VAS and ODI scores of OVCF patients treated by vertebral body augmentation were significantly improved (Table 3). On the 2nd day postoperation, VAS decreased to 3.42 ± 0.69 and ODI to 17.39 ± 4.99. At the last follow-up, VAS decreased to 1.69 ± 0.79 and ODI to 12.06 ± 4.18. These findings further indicated that VA could significantly relieve pain and improve the quality of life of patients. Clark et al.'s [20] findings showed that vertebral augmentation provides greater pain relief than conservative treatment for acute OVCFs. Klazen et al.'s [21] study also showed that vertebral reinforcement is superior to conservative treatment in pain relief. However, Firanescu et al.'s [22] randomized sham-controlled clinical trial reported that vertebral reinforcement did not show a statistically significant improvement in pain reduction over sham surgery for OVCFs in the acute phase. However, vertebral augmentation has the advantages of a rapid effect, easy operation, little damage, and a short operation time and wide use in clinical practice [23]. The mechanism of vertebral augmentation for pain relief remains controversial, and current research suggests that it may be related to the following factors [24]: (1) the operation provides corresponding mechanical strength and stability for the injured vertebra and relieves pain caused by the abnormal activity of the vertebral body; (2) the polymerization of bone cement produces a thermal effect, which burns the nerve tissue at the fractured end; (3) the chemical toxicity of bone cement itself causes nerve ending necrosis. In general, vertebral augmentation can rapidly relieve pain and restore functional activity by injecting bone cement to improve vertebral stability and reduce nerve ending stimulation, which is also the primary goal of OVCF treatment [25, 26].

### 4.2. Sufficient Dispersion of Bone Cement Is More Conducive to Reducing Postoperative Residual Pain

No significant difference was found in the preoperative scores between the groups through intergroup comparison analysis of the VAS and ODI scores (Table 1). However, the VAS and ODI scores of Group A were significantly better than those of Group B on the 2nd day postoperation and at the last follow-up. The present study showed that sufficient diffusion of bone cement within the fracture line is more conducive to reducing postoperative residual pain and improving patients' quality of life. Mo et al.'s [27] study found that an insufficient distribution of bone cement can lead to inadequate short- and medium-term postoperative pain relief, which may be related to the uneven distribution of bone cement leading to displacement of the fractured vertebral body and micromotion around the fracture [28]. Tan et al. [29] found that bone cement is evenly distributed and in close contact with the upper and lower endplates, leading to better maintenance of the strength and height of the vertebral body, a reduced risk of refracture, and improved chronic back pain in the patient.

In addition, other studies have found that residual pain after vertebral augmentation is associated with thoracolumbar fascia injury, erector spinae muscle spasm, intraoperative puncture injury, the bone cement injection volume, bone mineral density, vertebral body infection, adverse psychological and mental factors, and the bone cement volume ratio [30]. Therefore, in the present study, we analyzed the factors that might affect the efficacy of surgery, such as recovery of the injured vertebral height, the local kyphosis angle, bone mineral density, the vertebral body volume, the bone cement volume, and the bone cement volume ratio.

### 4.3. VA Can Raise the IVH and Reduce the LKA

After VA, IVH was higher than before; correspondingly, LKA was lower than before. The present study further confirmed the role of VA in restoring the height of the injured vertebra, reducing the LKA, and thereby improving the stability of the vertebra. However, we compared IVH and LKA between the two groups; no significant difference was found. This finding indicates that, in the present study, different distribution states of bone cement did not affect IVH and LKA. At the same time, different diffusion states of bone cement did not affect the relationship between IVH, LKA and postoperative residual pain. Vertebral augmentation can promote the height recovery of the injured vertebra and reduce the LKA in the short-term, which is conducive to reducing the pain of joints, muscles, and ligaments caused by spinal deformations [31, 32].

### 4.4. Sufficient Dispersion of Bone Cement Is Beneficial to Improve the Volume Ratio and Reduce Pain

In this study, no significant difference was found in the injection volume between the groups (*P*=0.122), among which Group A was 6.11 ± 1.65 ml and Group B was 5.15 ± 1.88 ml. The bone cement injection volume is an important factor affecting residual pain after vertebral augmentation. Some studies have found that an injection volume of bone cement >4.5 ml can achieve pain relief [33]. When the injection volume of bone cement is 5 ml to 6 ml, the overall treatment effect is more ideal [34].

The vertebral body volume was 29.86 ± 7.99 cm³ in Group A and 33.81 ± 9.27 cm³ in Group B, and the statistical analysis was *P*=0.187. No significant difference was found between the groups. However, the bone cement volume in Group A was higher than that in Group B, which was 7.08 ± 2.25 cm³ in Group A and 5.14 ± 1.60 cm³ in Group B (*P*=0.010). After calculation, the volume ratio of bone cement was 23.70% in Group A and 15.11% in Group B (*P* < 0.001). Studies have shown that the volume ratio of bone cement is positively correlated with pain relief. When the volume ratio of bone cement is greater than 27.8%, the analgesic effect is better [35]. In addition, a cement volume ratio of more than 40.5% increases the risk of adjacent vertebral fractures [36]. Thus, injecting a sufficient amount of bone cement and maintaining a sufficient dispersion state can increase the volume and volume ratio of bone cement and is more conducive to reducing postoperative residual pain.

### 4.5. Surgical Options

Regarding the surgical methods, vertebral augmentation mainly includes percutaneous vertebroplasty and kyphoplasty. We chose percutaneous unilateral extrapedicular balloon kyphoplasty, whose advantages are as follows [37–40]: first, kyphoplasty has more advantages in restoring the height of the injured vertebra and promoting the diffusion and distribution of bone cement in the fracture line. Second, unilateral puncture can shorten the operation time, and the external pedicle approach can also ensure better dispersion of bone cement. Third, kyphoplasty reduces the damage to important organs, spinal cord, nerves, vertebral body attachments, the dural sac, and the soft tissue caused by bilateral puncture.

In the present study, the diffusion of bone cement in the vertebral body was visually displayed. By establishing a three-dimensional model, the shape of bone cement in the diseased vertebra can be more intuitively analyzed, and the diffusion volume and volume ratio of bone cement can be accurately calculated. The present study demonstrated that the better is the dispersion of bone cement in the fracture line area, the better is the surgical effect. In summary, after determining the surgical plan, controlling the period of the operation, choosing the acute phase for the operation, controlling the amount of bone cement injected, injecting it in the bone cement toothpaste period, and ensuring the proportion of the bone cement volume in the vertebral body are all necessary to ensure the effect of the operation and improve clinical efficacy. At the same time, clarifying the mechanism of bone cement analgesia and finding more potential factors affecting residual pain are future research directions.

## 5. Limitations

Our study has limitations. This study was a single-center retrospective analysis with a time span of three years and a relatively small number of cases, and the data were easily available through the electronic medical record system. Therefore, we did not perform routine power or sample size calculations. Further large-scale, multifactor, and prospective studies must be performed.

## 6. Conclusions

Our study showed that bone cement has sufficient diffusion in the fracture line area, which is beneficial for relieving postoperative pain and improving quality of life.

## Figures and Tables

**Table 1:** Comparison between the groups.

	Group A (*n* = 23)	Group B (*n* = 13)	95% CI	*P* value
Age (years), mean ± SD (range)	76.30 ± 9.88 (63–98)	74.38 ± 7.29 (62–84)	−4.463 to 8.303	0.545
Sex (male/female), *n* (%)	2 (8)/21 (91)	0 (0)/13 (100)		0.274
Fracture segment				
Thoracic (T1-10)	5	2		
Thoracolumbar (T11-L2)	16	9		
Lumbar (L3-5)	2	2		
BMD (g/cm³), mean ± SD (range)	−2.84 ± 0.32 (−3.7 to −2.5)	−2.93 ± 0.41 (−3.9 to −2.5)	−0.161 to 0.336	0.480
Injection volume (ml), mean ± SD (range)	6.11 ± 1.65 (4.0–10.0)	5.15 ± 1.88 (3.0–9.0)	−0.268 to 2.177	0.122
Surgery time (min), mean ± SD (range)	30.87 ± 8.02 (23–60)	33.46 ± 8.43 (27–55)	−8.351 to 3.167	0.367
VAS (0–10), mean ± SD (range)				
Preoperation	7.13 ± 0.76 (6–9)	7.31 ± 0.86 (6–9)	−0.736 to 0.382	0.524
2nd day postoperation	3.17 ± 0.65 (2–5)	3.85 ± 0.56 (3–5)	−1.108 to −0.236	0.004
Last follow-up	1.35 ± 0.57 (0–2)	2.31 ± 0.75 (1–3)	−1.412 to −0.508	<0.001
ODI (0–100), mean ± SD (range)				
Preoperation	77.65 ± 4.58 (68–88)	75.23 ± 5.75 (64–88)	−1.120 to 5.963	0.174
2nd day postoperation	15.48 ± 4.14 (8–22)	20.77 ± 4.67 (14–28)	−8.346 to −2.236	0.001
Last follow-up	10.52 ± 3.78 (2–16)	14.77 ± 3.52 (10–20)	−6.847 to −1.648	0.002
IVH (mm), mean ± SD (range)				
Preoperation	16.32 ± 3.82 (9.52–22.31)	16.81 ± 3.21 (12.04–24.37)	−3.040 to 2.061	0.699
2nd day postoperation	18.74 ± 4.56 (11.63–28.49)	18.83 ± 3.75 (14.00–26.21)	−3.121 to 2.933	0.950
Last follow-up	18.87 ± 4.26 (13.56–26.56)	18.87 ± 3.47 (13.63–25.63)	−2.822 to 2.818	0.999
LKA (°), mean ± SD (range)				
Preoperation	18.44 ± 8.28 (3.19–34.90)	15.18 ± 10.41 (0.04–30.61)	−3.142 to 9.678	0.308
2nd day postoperation	14.89 ± 8.76 (2.82–32.45)	13.17 ± 7.99 (2.36–28.51)	−4.269 to 7.711	0.563
Last follow-up	16.11 ± 9.65 (3.65–36.32)	14.28 ± 8.68 (2.65–28.64)	−4.739 to 8.408	0.574
Vertebral body volume (cm³), mean ± SD (range)	29.86 ± 7.99 (15.96–43.97)	33.81 ± 9.27 (18.77–45.12)	−9.921 to 2.016	0.187
Bone cement volume (cm³), mean ± SD (range)	7.08 ± 2.25 (3.23–11.36)	5.14 ± 1.60 (2.61–7.27)	0.501 to 3.381	0.010
Volume ratio (%)	23.70	15.11		<0.001

Note: BMD, bone mineral density; VAS, visual analog scale; ODI, Oswestry disability index score; IVH, injured vertebral height; LKA, local kyphosis angle.

**Table 2:** VAS and ODI within-group comparisons.

		Mean ± SD (range)	95% CI	*P* value
Group A (*n* = 23)				
VAS (0–10)	Paired 1	7.13 ± 0.76 (6–9)	3.713–4.200	<0.001
		3.17 ± 0.65 (2–5)		
	Paired 2	3.17 ± 0.65 (2–5)	1.421–2.231	<0.001
		1.35 ± 0.57 (0–2)		
ODI (0–100)	Paired 1	77.65 ± 4.58 (68–88)	60.568–63.780	<0.001
		15.48 ± 4.14 (8–22)		
	Paired 2	15.48 ± 4.14 (8–22)	2.990–6.923	<0.001
		10.52 ± 3.78 (2–16)		

Group B (*n* = 13)				
VAS (0–10)	Paired 1	7.31 ± 0.86 (6–9)	2.992–3.931	<0.001
		3.85 ± 0.56 (3–5)		
	Paired 2	3.85 ± 0.56 (3–5)	1.008–2.068	<0.001
		2.31 ± 0.75 (1–3)		
ODI (0–100)	Paired 1	75.23 ± 5.75 (64–88)	50.572–58.351	<0.001
		20.77 ± 4.66 (14–28)		
	Paired 2	20.77 ± 4.66 (14–28)	2.546–9.454	0.003
		14.77 ± 3.52 (10–20)		

Note: paired sample *t*-test. Paired 1, preoperation was paired with the 2nd day postoperation. Paired 2 and 2nd day postoperation were paired with the last follow-up.

**Table 3:** Comparison of VAS and ODI before and after VA.

	Mean ± SD (range)	95% CI	*P* value
VAS (0–10), (*n* = 36)			
Paired 1	7.19 ± 0.79 (6–9)	3.547–4.008	<0.001
	3.42 ± 0.69 (2–5)		
Paired 2	3.42 ± 0.69 (2–5)	1.413–2.031	<0.001
	1.69 ± 0.79 (0–3)		

ODI (0–100), (*n* *=* 36)			
Paired 1	76.78 ± 5.09 (64–88)	57.331–61.447	<0.001
	17.39 ± 4.99 (8–28)		
Paired 2	17.39 ± 4.99 (8–28)	3.660–7.007	<0.001
	12.06 ± 4.18 (2–20)		

Note: paired sample *t*-test. Paired 1, preoperation was paired with the 2nd day postoperation. Paired 2 and 2nd day postoperation were paired with the last follow-up.

## Data Availability

The data used to support the findings of this study are available from the corresponding author upon request.

## Supplementary Materials

Supplementary Materials Supplementary file 1: raw data.
