# Supplementary material for: Correlation Analysis between Residual Pain after Vertebral Augmentation and the Diffusion Distribution of Bone Cement: A Retrospective Cohort Study
Source: Pain Res Manag. 2023 Jan 4;2023:1157611. doi: 10.1155/2023/1157611 (PMC9833933; doi:10.1155/2023/1157611)
Supplement: Supplementary Materials — Supplementary file 1: raw data. [file 1157611.f1.doc]

| Group | NAME | GENDER | AGE | Fracture segment | Surgery time(min) | Injection volume (ml) | Vertebral body volume | Bone cement volume | Volume ratio | BMD (g/cm³) | IVH (mm),Pre-operation | IVH (mm),2nd day post-operation | IVH (mm),Last follow-up | LKA (°),Pre-operation | LKA (°),2nd day post-operation | LKA (°),Last follow-up | ODI,Pre-operation | ODI,2nd day post-operation | ODI,Last follow-up | VAS,Pre-operation | VAS,2nd day post-operation | VAS,Last follow-up |
| --- | --- | --- | --- | --- | --- | --- | --- | --- | --- | --- | --- | --- | --- | --- | --- | --- | --- | --- | --- | --- | --- | --- |
| A | 01 | 2 | 77 | T9 | 30 | 5.0 | 18.897 | 3.449 | 0.1825 | -2.5 | 9.52 | 11.63 | 13.56 | 3.40 | 4.46 | 3.65 | 76 | 20 | 12 | 7 | 3 | 2 |
| A | 02 | 2 | 78 | L1 | 29 | 5.0 | 32.35 | 5.906 | 0.1826 | -3.7 | 15.57 | 15.16 | 14.86 | 24.10 | 10.99 | 16.36 | 76 | 14 | 12 | 7 | 3 | 2 |
| A | 03 | 2 | 75 | L3 | 30 | 5.0 | 39.812 | 7.663 | 0.1924 | -2.6 | 13.85 | 15.34 | 16.98 | 15.15 | 22.98 | 36.32 | 80 | 22 | 14 | 6 | 3 | 2 |
| A | 04 | 2 | 83 | T12 | 28 | 6.0 | 29.808 | 5.757 | 0.1931 | -2.7 | 9.56 | 14.38 | 13.56 | 34.90 | 32.45 | 32.65 | 76 | 16 | 16 | 7 | 4 | 1 |
| A | 05 | 2 | 63 | T8 | 25 | 4.0 | 15.962 | 3.233 | 0.2025 | -2.9 | 11.29 | 16.58 | 15.23 | 18.12 | 10.29 | 9.58 | 86 | 20 | 6 | 8 | 3 | 2 |
| A | 06 | 2 | 67 | L2 | 32 | 9.0 | 27.475 | 5.596 | 0.2037 | -3.1 | 20.42 | 27.21 | 26.56 | 17.12 | 14.29 | 13.26 | 78 | 16 | 6 | 8 | 4 | 1 |
| A | 07 | 2 | 66 | L1 | 23 | 7.0 | 30.972 | 6.436 | 0.2078 | -3.4 | 14.7 | 14.57 | 15.36 | 12.50 | 7.91 | 6.96 | 82 | 18 | 8 | 8 | 3 | 2 |
| A | 08 | 2 | 64 | T12 | 27 | 5.0 | 32.112 | 6.753 | 0.2103 | -2.9 | 13.48 | 16.70 | 15.96 | 15.68 | 17.41 | 15.26 | 76 | 18 | 8 | 7 | 3 | 1 |
| A | 09 | 2 | 83 | L1 | 25 | 7.5 | 40.724 | 9.010 | 0.2212 | -2.8 | 20.94 | 24.09 | 24.65 | 22.52 | 24.00 | 26.23 | 76 | 18 | 10 | 7 | 3 | 1 |
| A | 10 | 2 | 64 | T9 | 36 | 5.0 | 18.926 | 4.231 | 0.2236 | -3.1 | 14.67 | 16.09 | 17.56 | 13.34 | 4.35 | 5.69 | 84 | 20 | 12 | 6 | 3 | 1 |
| A | 11 | 2 | 98 | T11 | 42 | 7.0 | 21.648 | 5.031 | 0.2324 | -2.8 | 12.44 | 14.47 | 13.69 | 18.77 | 13.92 | 16.36 | 88 | 22 | 14 | 7 | 3 | 1 |
| A | 12 | 1 | 70 | T11 | 37 | 5.0 | 34.523 | 8.043 | 0.2330 | -2.5 | 17.7 | 17.77 | 16.89 | 15.73 | 6.69 | 7.89 | 76 | 18 | 12 | 7 | 3 | 2 |
| A | 13 | 2 | 70 | L1 | 26 | 5.0 | 25.651 | 6.070 | 0.2366 | -2.8 | 19.18 | 16.27 | 16.89 | 19.89 | 17.26 | 16.52 | 74 | 16 | 16 | 7 | 3 | 1 |
| A | 14 | 2 | 85 | L3 | 26 | 7.5 | 34.359 | 8.238 | 0.2398 | -2.8 | 21.3 | 25.38 | 26.30 | 25.07 | 28.30 | 28.63 | 78 | 14 | 12 | 7 | 2 | 1 |
| A | 15 | 2 | 87 | T8 | 33 | 5.0 | 19.113 | 4.730 | 0.2475 | -2.5 | 11.93 | 17.01 | 16.89 | 22.02 | 25.19 | 26.36 | 80 | 18 | 16 | 6 | 3 | 2 |
| A | 16 | 2 | 77 | L2 | 31 | 10.0 | 43.966 | 10.886 | 0.2476 | -3.1 | 20.98 | 28.49 | 23.69 | 3.19 | 3.20 | 4.59 | 80 | 10 | 10 | 8 | 4 | 1 |
| A | 17 | 2 | 65 | T12 | 23 | 5.0 | 29.667 | 7.486 | 0.2523 | -2.5 | 15.55 | 16.78 | 17.89 | 20.71 | 11.14 | 13.65 | 72 | 12 | 8 | 8 | 4 | 1 |
| A | 18 | 2 | 69 | L1 | 27 | 6.5 | 34.395 | 8.866 | 0.2578 | -2.5 | 19.12 | 20.52 | 21.03 | 28.44 | 26.46 | 24.98 | 76 | 10 | 2 | 7 | 3 | 0 |
| A | 19 | 2 | 81 | L1 | 26 | 4.5 | 35.285 | 9.522 | 0.2699 | -3.1 | 18.45 | 22.64 | 23.63 | 15.69 | 14.51 | 13.59 | 74 | 12 | 14 | 7 | 3 | 2 |
| A | 20 | 2 | 89 | T12 | 36 | 5.0 | 30.071 | 8.126 | 0.2702 | -3.1 | 22.31 | 24.81 | 25.69 | 8.31 | 2.82 | 3.65 | 68 | 8 | 12 | 7 | 3 | 2 |
| A | 21 | 1 | 89 | T12 | 60 | 9.0 | 39.825 | 11.357 | 0.2852 | -2.9 | 18.04 | 19.16 | 18.96 | 34.53 | 23.81 | 26.36 | 78 | 12 | 6 | 7 | 3 | 1 |
| A | 22 | 2 | 85 | T12 | 33 | 7.5 | 32.942 | 9.969 | 0.3026 | -2.6 | 18.02 | 18.93 | 20.36 | 24.79 | 10.95 | 11.65 | 72 | 10 | 6 | 9 | 5 | 1 |
| A | 23 | 2 | 70 | T9 | 25 | 5.0 | 18.247 | 6.500 | 0.3562 | -2.5 | 16.35 | 16.94 | 17.89 | 10.24 | 9.11 | 10.36 | 80 | 12 | 10 | 6 | 2 | 1 |
| B | 01 | 2 | 72 | L1 | 29 | 4.0 | 27.542 | 3.650 | 0.1325 | -2.7 | 20.21 | 20.62 | 20.36 | 5.45 | 5.07 | 2.65 | 74 | 22 | 12 | 8 | 4 | 3 |
| B | 02 | 2 | 62 | T12 | 30 | 4.5 | 31.265 | 4.243 | 0.1357 | -3.1 | 16.6 | 18.13 | 19.36 | 11.13 | 8.00 | 15.56 | 78 | 24 | 14 | 8 | 4 | 2 |
| B | 03 | 2 | 84 | L3 | 36 | 7.0 | 32.784 | 4.516 | 0.1378 | -2.8 | 15.98 | 17.01 | 16.98 | 2.29 | 5.26 | 5.63 | 64 | 28 | 20 | 7 | 3 | 2 |
| B | 04 | 2 | 84 | T8 | 30 | 3.0 | 18.770 | 2.613 | 0.1392 | -2.6 | 13.24 | 14.30 | 13.63 | 7.33 | 10.04 | 9.63 | 88 | 26 | 18 | 7 | 4 | 2 |
| B | 05 | 2 | 80 | L2 | 48 | 6.0 | 43.354 | 6.079 | 0.1402 | -3.4 | 12.04 | 14.00 | 13.69 | 22.71 | 12.61 | 16.35 | 78 | 22 | 16 | 7 | 4 | 1 |
| B | 06 | 2 | 80 | L4 | 30 | 9.0 | 45.122 | 6.483 | 0.1437 | -2.7 | 15.96 | 20.39 | 19.63 | 22.39 | 25.13 | 26.96 | 68 | 14 | 14 | 8 | 5 | 3 |
| B | 07 | 2 | 69 | T12 | 28 | 4.0 | 24.143 | 3.603 | 0.1492 | -3.3 | 15.9 | 16.26 | 16.78 | 23.43 | 13.82 | 19.63 | 78 | 18 | 18 | 6 | 3 | 2 |
| B | 08 | 2 | 76 | L1 | 33 | 3.0 | 44.725 | 6.767 | 0.1513 | -2.7 | 15.13 | 19.00 | 20.36 | 22.27 | 18.52 | 15.15 | 78 | 22 | 16 | 7 | 4 | 3 |
| B | 09 | 2 | 78 | T10 | 27 | 3.0 | 25.559 | 3.934 | 0.1539 | -3.9 | 15.27 | 20.63 | 19.63 | 30.61 | 28.51 | 28.64 | 76 | 16 | 12 | 8 | 4 | 2 |
| B | 10 | 2 | 69 | L1 | 30 | 5.0 | 42.824 | 7.044 | 0.1645 | -2.5 | 16.81 | 18.17 | 19.56 | 18.39 | 12.28 | 11.56 | 72 | 14 | 20 | 9 | 4 | 1 |
| B | 11 | 2 | 75 | T11 | 55 | 5.0 | 23.732 | 3.947 | 0.1663 | -2.6 | 16.74 | 15.13 | 16.13 | 26.97 | 20.77 | 23.65 | 76 | 24 | 10 | 7 | 4 | 3 |
| B | 12 | 2 | 62 | L2 | 30 | 6.0 | 38.547 | 6.671 | 0.1730 | -3.1 | 20.28 | 24.94 | 23.63 | 0.04 | 8.84 | 6.89 | 72 | 16 | 12 | 6 | 4 | 3 |
| B | 13 | 2 | 76 | L2 | 29 | 7.5 | 41.163 | 7.271 | 0.1766 | -2.7 | 24.37 | 26.21 | 25.63 | 4.28 | 2.36 | 3.29 | 76 | 24 | 10 | 7 | 3 | 3 |
